# Measles Reemergence in Ceará, Northeast Brazil, 15 Years after Elimination

**DOI:** 10.3201/eid2109.150391

**Published:** 2015-09

**Authors:** Robério D. Leite, Juliana L.T.M.S. Barreto, Anastácio Q. Sousa

**Affiliations:** Hospital São José de Doenças Infecciosas, Fortaleza (R.D. Leite, J.L.T.M.S. Barreto, A.Q. Sousa);; Universidade Federal do Ceará, Fortaleza, Brazil (R.D. Leite, A.Q. Sousa)

**Keywords:** measles, outbreak, endemic diseases, viruses, Brazil

**To the Editor:** Measles was endemic in Brazil before 2000 and caused large outbreaks every 2 or 3 years (*1*). Although measles was eliminated in Brazil in 2000, cases have continued to be imported (*2*,*3*). During 2001–2014, the median annual number of measles cases reported in Brazil was 50 (range 2–712). The median annual number of Brazilian states with reported cases was 2.5 (range 1–7). Since elimination, the highest numbers of cases reported in Brazil occurred in 2013 (220) and in 2014 (712) (*3*–*5*). According to the Pan American Health Organization, endemic transmission is reestablished when epidemiologic and laboratory evidence indicate that a chain of transmission of a virus strain has continued uninterrupted for >12 months in a defined geographic area (*6*).

From December 2, 2013, through December 31, 2014, in the state of Ceará, Brazil, 681 measles cases were reported. A measles case was considered confirmed when a patient exhibited fever, rash, and >1 of 3 symptoms and signs (i.e., cough, runny nose, conjunctivitis); was positive for IgM and negative for IgG against measles virus; and had not been vaccinated in the previous 21 days. D8 genotype, the same virus genotype that was circulating in Europe, was the only genotype identified, and how the virus was introduced into the region was not clear (*4*,*5*). From 2000 to 2013, vaccine coverage among children 12 months of age remained >95% in Ceará, although that coverage was not homogeneous for the whole state. In 14.7% (27/184) of municipalities, the vaccination coverage was much lower (*4*). Pernambuco, the state that borders southern Ceará, reported a measles outbreak with 222 confirmed cases from March 2013 through March 2014 (*4*,*5*,*7*). Thus, the timing of the 2 outbreaks overlapped. 

During December 2013–December 2014, Ceará’s outbreak seemed to evolve in 2 waves: the first from epidemiologic weeks 3 through 6 (mainly in Fortaleza, the capital of Ceará) and the second from epidemiologic weeks 27 through 53 (mainly on the northwest side of Ceará, an economically disadvantaged region, which also included the capital). Cases were confirmed in 15.8% (29/184) of all municipalities. Most patients (47.3%; 322) were from Fortaleza, followed by Massapê (18.6%; 127) and Sobral (12.2%; 83) (Figure). 

**Figure:**
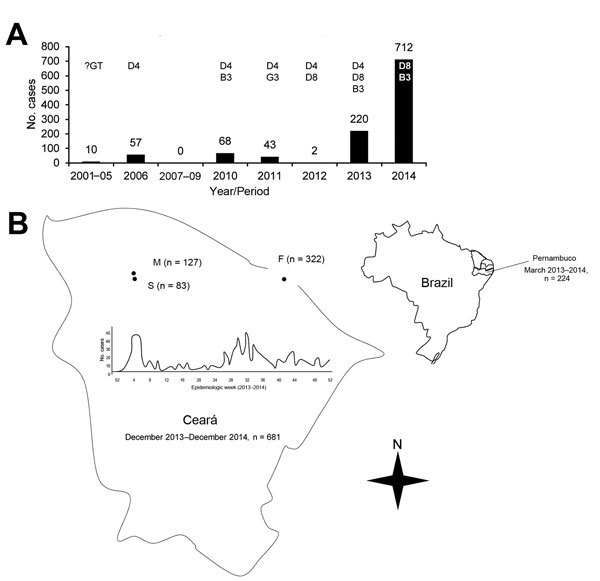
Measles cases reported in Brazil after elimination, 2001–2014. A) Cases and genotypes identified, by year. B) Spatial distribution of measles outbreaks in the states of Pernambuco and Ceará during 2013–2014, in which only genotype D8 was identified. Genotypes B3 and D4, observed during 2013–2014, were reported in other Brazilian states. The cities with the highest number of cases are highlighted on the map, as well as the evolution of its outbreak, which had 2 waves with peaks in the first and second halves of 2014. Data through December 31, 2014. F, Fortaleza; M, Massapê; S, Sobral; B3 , genotype B3; D4, genotype D4; D8, genotype D8; G3, genotype G3; ?GT, unknown genotype. Sources: (*3**,**5**,**7*).

Children <12 months of age were the most affected group (27.5%; 187), followed by patients 20–29 years (19.2%; 131) and those 15–19 years (14.4%; 98). The age distribution was significantly different between Fortaleza and the 2 inner cities (together), with more cases reported among those <12 months of age (37.6% [121/322] vs. 14.3% [30/210], respectively) and for those 15–29 years (25.2% [81/322] vs. 43.8% [92/210], respectively) (p<0.001 for both comparisons) (*5*). Vaccination status of affected patients (data through August 8, 2014) was the following: unvaccinated, 22.2% (55/252) <1 year of age and 31.3% (79/252) >1 year of age; unknown vaccination status, 27.4% (69/252); and received only 1 dose of vaccine, 18.7% (47/252) (*8*). No deaths were reported (*4*). The main reported symptoms were rash (100%), fever (100%), cough (84.5%), runny nose (68.2%), and conjunctivitis (60.3%) (*8*). 

Response vaccination activities have taken 10–20 weeks to be initiated in some municipalities after the first cases were recognized. Vaccination campaigns involving children 6–60 months of age are being intensified and surveillance for suspected cases has increased, but as of January 1, 2015, the chain of transmission appeared ongoing (*4*,*5*). In addition, one cannot underestimate the fact that health professionals in Ceará had not seen cases of measles for 15 years. Younger health professionals had never seen even 1 case, and this lack of familiarity may have had some effect on surveillance, rapid recognition of new cases, and adoption of control measures. This difficulty of recognition should be taken into account in regions that have been free of endemic measles transmission for many years. 

In conclusion, the measles outbreak in Ceará was probably imported directly from Europe or from there through the bordering state of Pernambuco (*4*,*5*,*9*). Cases were concentrated in Fortaleza and the northwest region of the state. Patient age distribution was significantly different between the capital, where the infection most affected children <12 months of age, and the inner cities, where it most affected persons 15–29 years of age. Current heterogeneous measles vaccine coverage (*4*,*5*); a delayed response and insufficient vaccination coverage in the past, particularly in socially disadvantaged populations from the inner cities; and difficulties in the prompt recognition and surveillance of suspected cases may explain why this outbreak occurred in a population with a vaccine coverage historically >95%. In addition, vaccination campaigns directed at children <5 years of age may not have been sufficient to interrupt the outbreak because a substantial number of older persons were susceptible. Most notably, because it has lasted >12 months, Ceará’s current outbreak may represent the reestablishment of endemic transmission of measles in the Americas.
